# Genome-wide association studies for methane emission and ruminal volatile fatty acids using Holstein cattle sequence data

**DOI:** 10.1186/s12863-020-00953-0

**Published:** 2020-11-23

**Authors:** Ali Jalil Sarghale, Mohammad Moradi Shahrebabak, Hossein Moradi Shahrebabak, Ardeshir Nejati Javaremi, Mahdi Saatchi, Majid Khansefid, Younes Miar

**Affiliations:** 1 Department of Animal Science, College of Agriculture and Natural Resources, University of Tehran, Karaj, 31587-11167 Iran; 2 Department of Animal Science and Aquaculture, Dalhousie University, Truro, B2N 5E3 Canada; 3 Department of Animal Science, Iowa State University, 806 Stange Road, Ames, IA 50011 USA; 4 American Simmental Association, Bozeman, MT 59715 USA; 5 Agriculture Victoria, AgriBio Centre for AgriBioscience, Bundoora, VIC 3083 Australia

**Keywords:** Methane emission, Whole-genome sequence, Iranian Holstein cattle, Genome-wide association study, Volatile fatty acids

## Abstract

**Background:**

Methane emission by ruminants has contributed considerably to the global warming and understanding the genomic architecture of methane production may help livestock producers to reduce the methane emission from the livestock production system. The goal of our study was to identify genomic regions affecting the predicted methane emission (PME) from volatile fatty acids (VFAs) indicators and VFA traits using imputed whole-genome sequence data in Iranian Holstein cattle.

**Results:**

Based on the significant-association threshold (*p* < 5 × 10^− 8^), 33 single nucleotide polymorphisms (SNPs) were detected for PME per kg milk (*n* = 2), PME per kg fat (*n* = 14), and valeric acid (*n* = 17). Besides, 69 genes were identified for valeric acid (*n* = 18), PME per kg milk (*n* = 4) and PME per kg fat (*n* = 47) that were located within 1 Mb of significant SNPs. Based on the gene ontology (GO) term analysis, six promising candidate genes were significantly clustered in organelle organization (GO:0004984, *p* = 3.9 × 10^− 2^) for valeric acid, and 17 candidate genes significantly clustered in olfactory receptors activity (GO:0004984, *p* = 4 × 10^− 10^) for PME traits. Annotation results revealed 31 quantitative trait loci (QTLs) for milk yield and its components, body weight, and residual feed intake within 1 Mb of significant SNPs.

**Conclusions:**

Our results identified 33 SNPs associated with PME and valeric acid traits, as well as 17 olfactory receptors activity genes for PME traits related to feed intake and preference. Identified SNPs were close to 31 QTLs for milk yield and its components, body weight, and residual feed intake traits. In addition, these traits had high correlations with PME trait. Overall, our findings suggest that marker-assisted and genomic selection could be used to improve the difficult and expensive-to-measure phenotypes such as PME. Moreover, prediction of methane emission by VFA indicators could be useful for increasing the size of reference population required in genome-wide association studies and genomic selection.

## Background

Methane is the second most abundant global anthropogenic greenhouse gas behind carbon dioxide [1]. The ruminant production system has considerable contribution to climate change by emitting methane, either directly from enteric fermentation or indirectly from feed production activities, deforestation, manure, etc. [2]. The majority of enteric methane (87%) is produced in the rumen [3] by the activity of methanogenic archaea under anaerobic conditions [2]. Traditionally, several strategies such as diet manipulations, chemo-genomics, chemical and biological feed additives and anti-methanogen vaccines, have been designed to mitigate methane emissions besides improving the efficiency of production system. However, a fundamental problem is that the ruminal microbiota is able to adapt rapidly to intervention strategies [4].

The amount of enteric-methane emissions from ruminants is affected by a variety of factors including feed intake and composition, fermentation process e.g. passage rate and rumen volume, physiological state of the animal, and variation among individual animals [4–6]. Reducing the methane produced by cattle through genetic selection is a highly desirable approach compared with other strategies (e.g. adding supplements such as nitrates to feed) because genetic improvement is heritable and cumulative across generations. De Haas et al. [7] reported that the estimated heritability of predicted methane emission (PME) per milk production and PME per milk production corrected for fat and protein content were 0.35 and 0.58, respectively. Moreover, genome-wide association studies (GWAS) have identified several single nucleotide polymorphisms (SNPs) associated with measured or predicted rate of methane emission in Holstein cattle [7] and Angus cattle [8]. Thus, genetic selection can be employed to reduce PME per unit of milk production. However, routine measurement of methane production is difficult and expensive in farm, and identified SNPs associated with methane production can only explain a small proportion of its genetic variation [9]. Nowadays, via the availability of sequence data that potentially consists of causative mutations, the prediction accuracy of genomic selection and resolution of GWAS can be improved [10]. Therefore, the goal of our study was to identify genomic regions affecting the predicted methane emission (PME) from volatile fatty acids (VFAs) indicators and VFA traits using imputed whole-genome sequence data in Iranian Holstein cattle.

## Results

The descriptive statistics for studied traits are presented in Table 1. In the least squares analysis of the studied traits, the contemporary group effect was statistically significant (*p* < 0.05) for PME, butyric acid and isovaleric acid, and the effect of age was only significant for PME (*p* < 0.05).

**Table 1:** Descriptive statistics for predicted methane emission (PME), PME per kg milk, PME per kg fat, and volatile fatty acids traits in Iranian Holstein cattle

Trait	Number	Mean	Range	Standard deviation	CV (%)
PME (ml)	146	14819.88	8564–19315	2235.15	15.08
PME per kg milk (ml)	146	384.42	316.5–478	31.50	8.19
PME per kg fat (ml)	146	15390.92	8556–31503	3340.50	21.70
Milk estimated breeding value	150	870.22	− 1299.20-2475.05	903.41	103.81
Acetic acid (%)	146	53.71	44.39–60.02	3.15	5.87
Propionic acid (%)	146	22.37	17.10–29.23	2.40	10.74
Butyric acid (%)	146	16.42	12.28–21.66	1.73	10.56
Valeric acid (%)	147	3.24	1.53–6.16	0.60	19.97
Isovaleric acid (%)	147	4.25	2.12–9.57	1.39	32.77

### Genome-wide association studies for volatile fatty acids

After removing the genotypes with low imputation accuracy (*R*^*2*^ < 0.30) and other criteria (e.g. call rate < 95%, MAF < 0.05, and Chi-square < 10^− 6^ of Hardy-Weinberg equilibrium), a total of 6,583,595 SNPs on 29 *Bos taurus* autosomes (BTAs) were remained. The number of quality-control passed imputed variants used for association analyses ranged from 125,756 on BTA25 to 443,774 on BTA1.

**Fig. 1:**
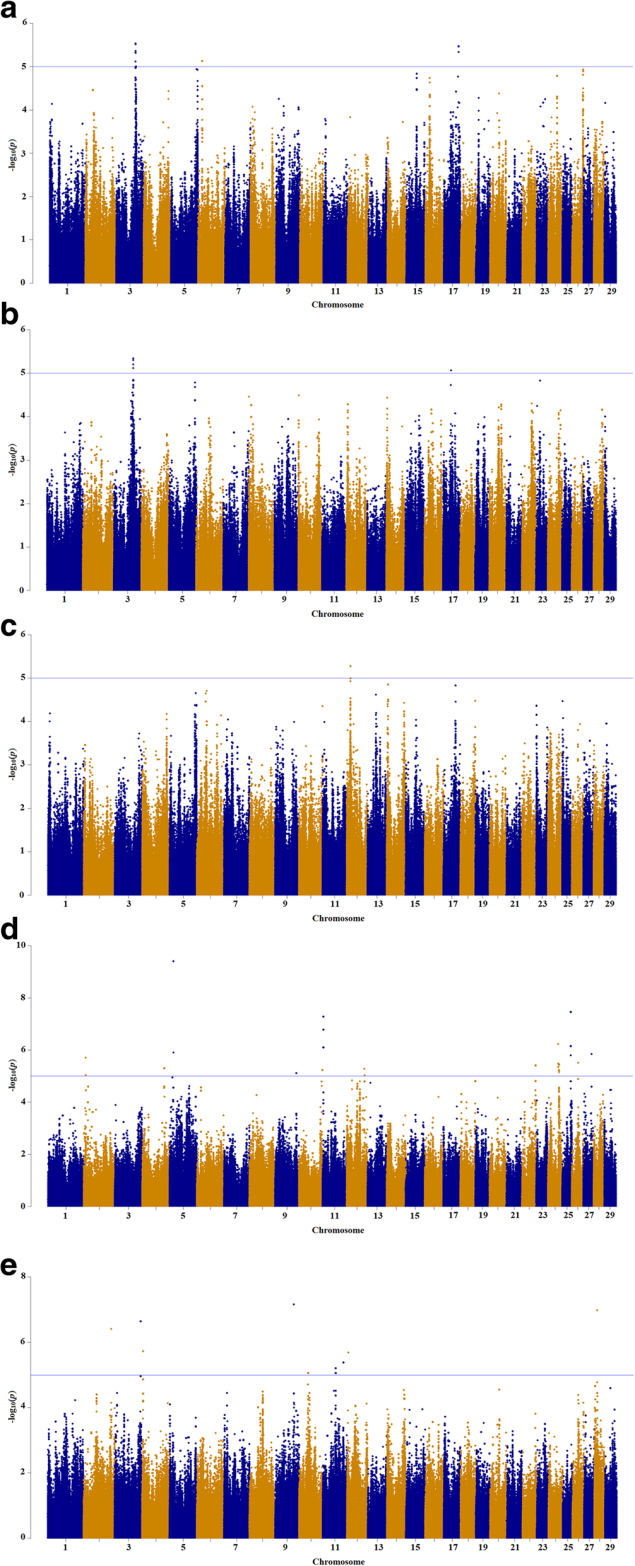
Manhattan plot of the genome-wide *p* values of association for VFA traits: **a** acetic acid; **b** propionic acid; **c** butyric acid; **d** valeric acid; **e** isovaleric acid. The solid line represents the *p* < 10^− 5^ significance threshold

The results demonstrated that 76 (on BTA3, 6, and 17), 3 (on BTA12), 5 (on BTA3 and 17), 274 (on BTA2, 4, 5, 9, 10, 11, 12, 22, 24, 25, 26 and 27) and 285 (BTA2, 3, 4, 9, 10, 11, 12 and 28) SNPs passed the suggestive-association threshold (*p* < 10^− 5^) for acetic acid (%), butyric acid (%), propionic acid (%), isovaleric acid (%) and valeric acid (%) traits, respectively (Fig. 1). Further, using the chromosome-wide Bonferroni correction threshold, we found two SNPs significantly associated with isovaleric acid (BTA9 and 28) and 29 SNPs with valeric acid (BTA5, 11, and 25). However, we did not discover any significant association for other VFA traits based on chromosome-wide Bonferroni correction. Moreover, using the recommended significant threshold of 5 × 10^− 8^, 18 SNPs (two on BTA5 and 16 on BTA 25) were associated with valeric acid variation, two on BTA5 found to be significant at the genome-wide Bonferroni correction threshold of 7.59 × 10^− 9^ (Table 2).

**Table 2:** Characteristics of most significant single nucleotide polymorphisms (SNPs) in significant regions based on 5 × 10^− 8^ threshold

Chromosome	SNP	Trait	Position (bp)	Allele substitution effect	S.E.	*P*-value
5	5:16795260	Valeric acid	16795260	−1.91	0.28	3.84e-10
25	25:37967076	Valeric acid	37967076	−1.30	0.22	3.42e-08
15	15:25797132	PME per kg milk	25797132	−22.66	3.93	5.00e-08
4	4:115131249	PME per kg fat	115131249	−10183.45	1484.85	2.14e-10
19	19:24494923	PME per kg fat	24494923	−13241.11	2120.06	4.89e-09
28	28:21771233	PME per kg fat	21771233	− 4709.37	811.77	4.30e-08
13	13:81673732	PME per kg fat	81673732	−10135.38	1745.10	4.17e-08

### Genome-wide association studies for predicted methane emission

**Fig. 2:**
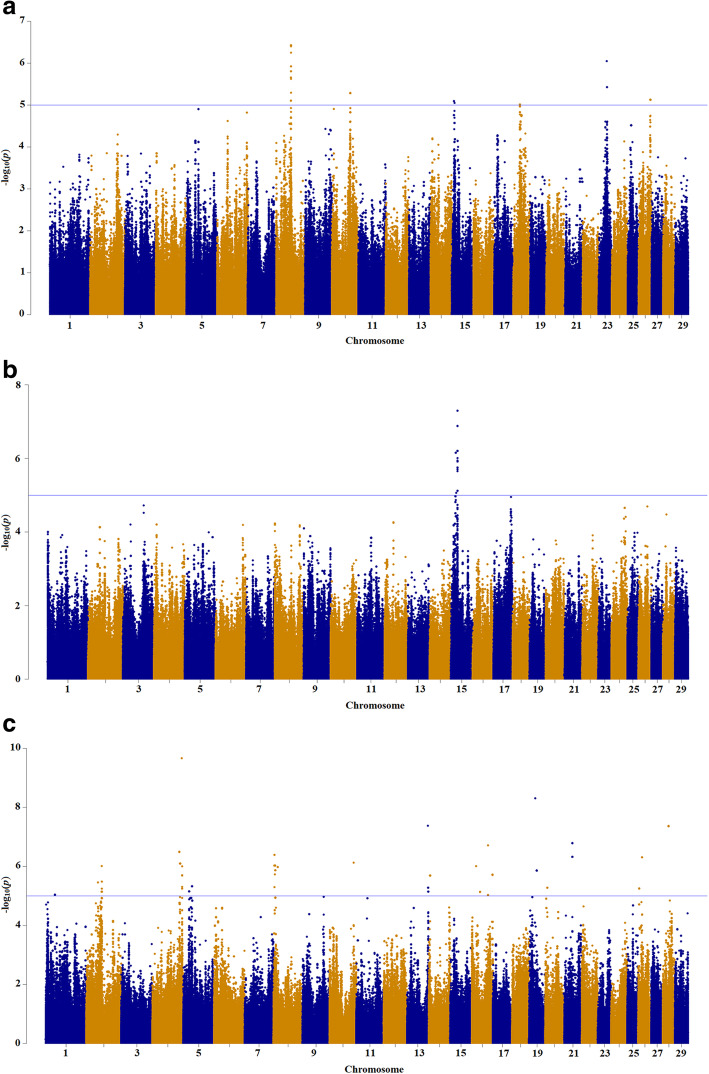
Manhattan plot of the genome-wide *p* values of association for PME traits: **a** PME; **b** PME per kg milk; **c** PME per kg fat. The solid line represents *p* < 1 × 10^− 5^ significance threshold

The GWAS results for PME, PME per kg milk, and PME per kg fat are presented in Manhattan plots in Fig. 2. According to suggestive regions (*p* < 10^− 5^), we found 53 SNPs (BTA8, 10, 15, 18, 23, and 26) for PME; 41 SNPs (BTA15) for PME per kg milk; and 308 SNPs (BTA1, 2, 4, 5, 8, 10, 13, 14, 16, 19, 20, 21, 26, and 28) for PME per kg fat. Although, the association studies did not show any significant SNPs for PME trait, the chromosome-wide Bonferroni correction identified five significant SNPs on BTA15 for PME per kg milk, 20 significant SNPs on BTA4, 13, 19, and 21 for PME per kg fat. PME per animal that were not adjusted per unit of product showed a decreased variation compared to the adjusted PME per kg of milk or fat i.e. two animals with an equal rate of methane emission do not necessarily have an equal efficiency for methane production (methane emission per unit of product). As a result, the power of the association studies to capture quantitative trait loci (QTL) without considering the adjustment of PME per unit of product can be diminished. Using the recommended Bonferroni correction threshold (*p* < 5 × 10^− 8^), we identified two SNPs on BTA15 for PME per kg milk, 14 SNPs on BTA4, 13, 19, and 28 for PME per kg fat (Table 2); and two SNPs on BTA4 and 19 passed the genome-wide Bonferroni correction for PME per kg fat (Table 2).

### Post-GWAS bioinformatics analysis

The candidate genes were identified within 1 Mb of most significant SNPs based on *p* < 5 × 10^− 8^. For valeric acid, two and 16 candidate genes were discovered around 5:16795260 and 25:37967076 SNPs, respectively (Table 3). For PME per kg milk, four candidate genes around 15:25797132 SNP, and for PME per kg fat 11, 3, 32 and 1 candidate genes were discovered around 4:115131249, 13:81673732, 19:24494923 and 28:21771233 SNPs, respectively (Table 4). Ten genes of 17 candidate genes for valeric acid remained for Gene Ontology (GO) term analysis as known and non-ambiguous genes. Based on the GO terms, six promising candidate genes were significantly clustered in organelle organization (GO:0004984, *p* = 3.9 × 10^− 2^) including *BAIAP2L1*, *SMURF1*, *ARPC1A*, *ARPC1B*, *BHLHA15* and *TRRAP*. For methane emission trait, twenty-five genes of 40 candidate genes remained for GO term analysis as known and non-ambiguous genes. Based on the GO terms, 17 candidate genes were significantly clustered in olfactory receptors activity (GO:0004984, *p* = 4 × 10^− 10^) including *LOC538966*, *LOC522582*, *LOC540082*, *LOC618593*, *LOC508980*, *LOC509525*, *LOC509526*, *LOC617122*, *LOC532238*, *OR1E1*, *LOC618124*, *OR1G1*, *LOC511509*, *LOC526294*, *LOC615901*, *LOC618112* and *LOC101902679*.

**Table 3:** The candidate or nearest genes to the most significant single nucleotide polymorphisms (SNPs) in significant regions based on 5 × 10^−8^ threshold for valeric acid trait

SNP name	SNP position	Ensembl gene ID	Gene start	Gene end	Gene name
5:16795260	16795260	ENSBTAG00000046197	16534698	16534778	MGAT4C^a^
		ENSBTAG00000020784	17207296	17208334	PTGR2^a^
25:37967076	37967076	ENSBTAG00000040568	37431139	37469998	ZNF789^a^
		ENSBTAG00000020440	37513721	37524164	LOC101910000
		ENSBTAG00000002090	37500102	37512370	CPSF4
		ENSBTAG00000020439	37524461	37532400	BUD31
		ENSBTAG00000044040	37532740	37544696	PDAP1
		ENSBTAG00000046248	37546485	37559078	ARPC1B
		ENSBTAG00000004242	37564390	37587556	ARPC1A
		ENSBTAG00000015737	37664542	37691692	KPNA7
		ENSBTAG00000007118	37777779	37801407	SMURF1
		ENSBTAG00000007113	37807383	37895047	TRRAP
		ENSBTAG00000005679	37902689	37918783	TMEM130
		ENSBTAG00000045896	38041960	38053172	NPTX2
		ENSBTAG00000019181	38227396	38280018	BAIAP2L1
		ENSBTAG00000022825	38281392	38287463	BRI3
		ENSBTAG00000046010	38342493	38343056	BHLHA15
		ENSBTAG00000004135	38348134	38395453	LMTK2

^a^unknown genes in cow, the names in the table are their orthologues in other species

**Table 4:** The candidate or nearest genes to the most significant single nucleotide polymorphisms (SNPs) in significant regions based on 5 × 10^− 8^ for predicted methane emission (PME) per kg milk and fat traits

Trait	SNP name	SNP position	Ensembl gene ID	Gene start	Gene end	Gene name
Methane per kg fat	4:115131249	115131249	ENSBTAG00000046461	114631249	115631249	SUMF1^a^
			ENSBTAG00000014372	114680160	114615444	SMARCD3
			ENSBTAG00000004540	114725191	114689767	NUB1
			ENSBTAG00000006232	114755250	114730382	WDR86
			ENSBTAG00000003019	114779653	114773449	CRYGN
			ENSBTAG00000031861	114855282	114803157	RHEB
			ENSBTAG00000002917	114925565	114885501	PRKAG2
			ENSBTAG00000045535	115301688	115246102	GALNTL5
			ENSBTAG00000021260	115360689	115335971	GALNT11
			ENSBTAG00000024199	115524670	115368063	KMT2C
			ENSBTAG00000014871	115631329	115629803	CCT8L2
	13:81673732	81673732	ENSBTAG00000007917	81601021	81604089	TSHZ2
			ENSBTAG00000030556	81854047	81863004	ZNF217
			ENSBTAG00000000835	82162091	82259990	BCAS1
	19:24494923	24494923	ENSBTAG00000008324	24481721	24480297	LOC618593
			ENSBTAG00000017440	24023406	23961074	METTL16
			ENSBTAG00000016806	24158415	24088805	PAFAH1B1
			ENSBTAG00000011786	24181331	24168990	CLUH
			ENSBTAG00000020000	24452796	24395974	RAP1GAP2
			ENSBTAG00000039018	24507032	24506058	OR1G1
			ENSBTAG00000040362	24521504	24520572	LOC540082
			ENSBTAG00000039411	24528384	24527446	LOC532238
			ENSBTAG00000039633	24539905	24538976	LOC522582
			ENSBTAG00000048282	24599226	24598261	LOC509525
			ENSBTAG00000048099	24613735	24612770	LOC509526
			ENSBTAG00000047049	24632514	24631549	LOC617122
			ENSBTAG00000026859	24655261	24654314	LOC508980
			ENSBTAG00000018221	24669376	24668411	LOC101902679
			ENSBTAG00000046652	24677969	24677022	LOC538966
			ENSBTAG00000037529	24708485	24707451	OR3A2^a^
			ENSBTAG00000038059	24712313	24711369	OR1E1
			ENSBTAG00000046960	24724257	24723313	LOC615901
			ENSBTAG00000045667	24731754	24730810	LOC618124
			ENSBTAG00000046217	24750164	24749223	LOC511509
			ENSBTAG00000047458	24756645	24755704	LOC618112
			ENSBTAG00000045899	24763240	24762296	LOC526294
			ENSBTAG00000032457	24802537	24801593	OR3A3^a^
			ENSBTAG00000015763	24824295	24809409	SPATA22
			ENSBTAG00000003629	24847108	24825866	ASPA
			ENSBTAG00000000020	24885437	24854533	TRPV3
			ENSBTAG00000018880	24913053	24890751	TRPV1
			ENSBTAG00000000829	24951464	24933750	SHPK
			ENSBTAG00000000831	24972141	24951988	CTNS
			ENSBTAG00000000833	24977062	24971932	TAX1BP3
			ENSBTAG00000025121	24978133	24977284	EMC6
			ENSBTAG00000015258	24996301	24983919	P2RX5
	28:21771233	21771233	ENSBTAG00000036111	21868711	21867759	AP2M1
PME per kg milk	15:25797132	25797132	ENSBTAG00000005843	25304051	25293299	REXO2
			ENSBTAG00000005945	25434051	25419219	NXPE4
			ENSBTAG00000031362	25524857	25515542	NXPE2
			ENSBTAG00000000977	26451509	26101411	CADM1

^a^unknown genes in cow, the names in the table are their orthologues in other species

**Fig. 3:**
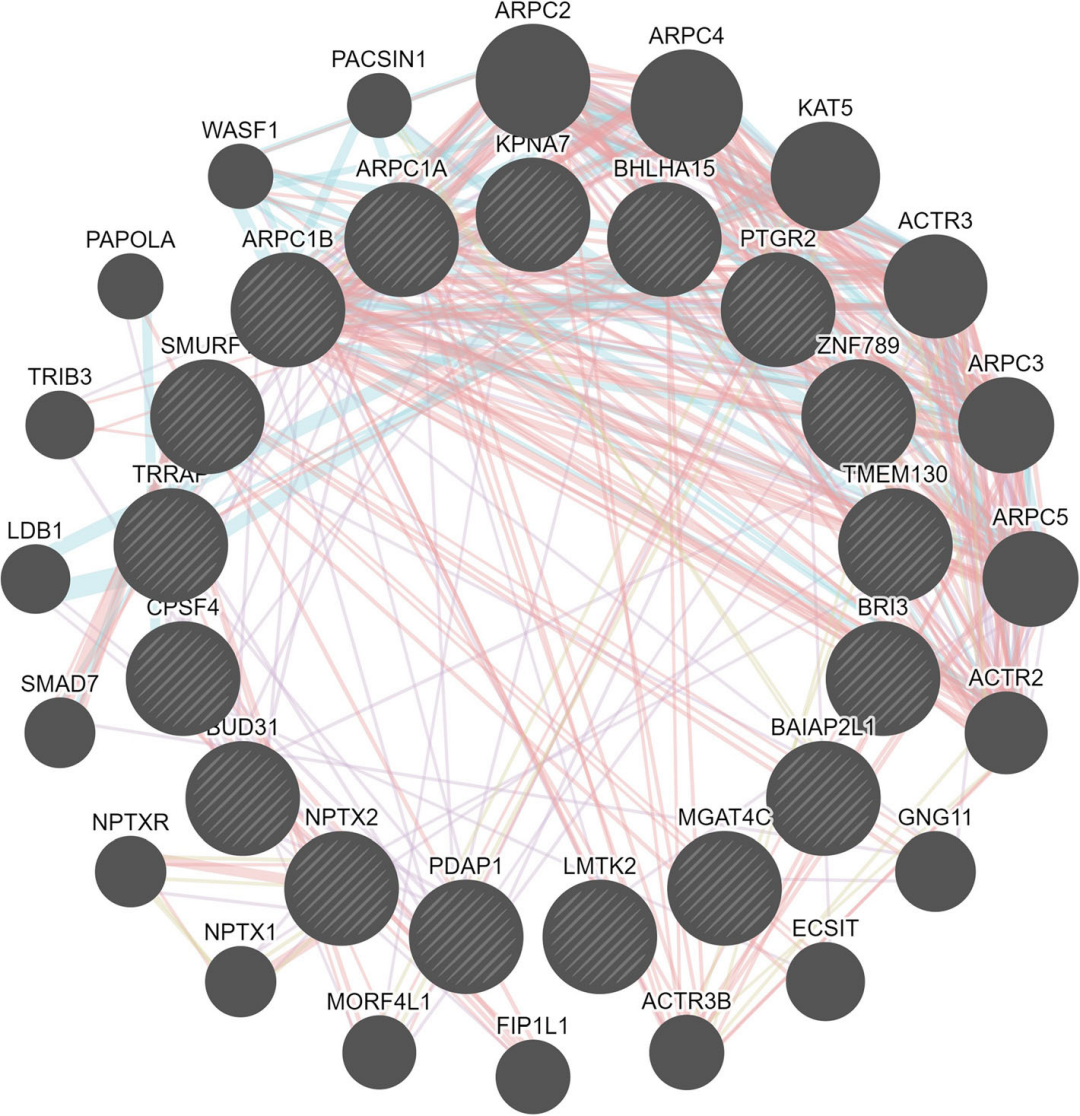
Gene networks analysis for valeric acid trait. Dark circles with and without slash represent candidate genes and associated genes, respectively. Arrows in pink, blue, red and bone color represent co-expression, pathway, physical interactions and shared protein domains, respectively

**Fig. 4:**
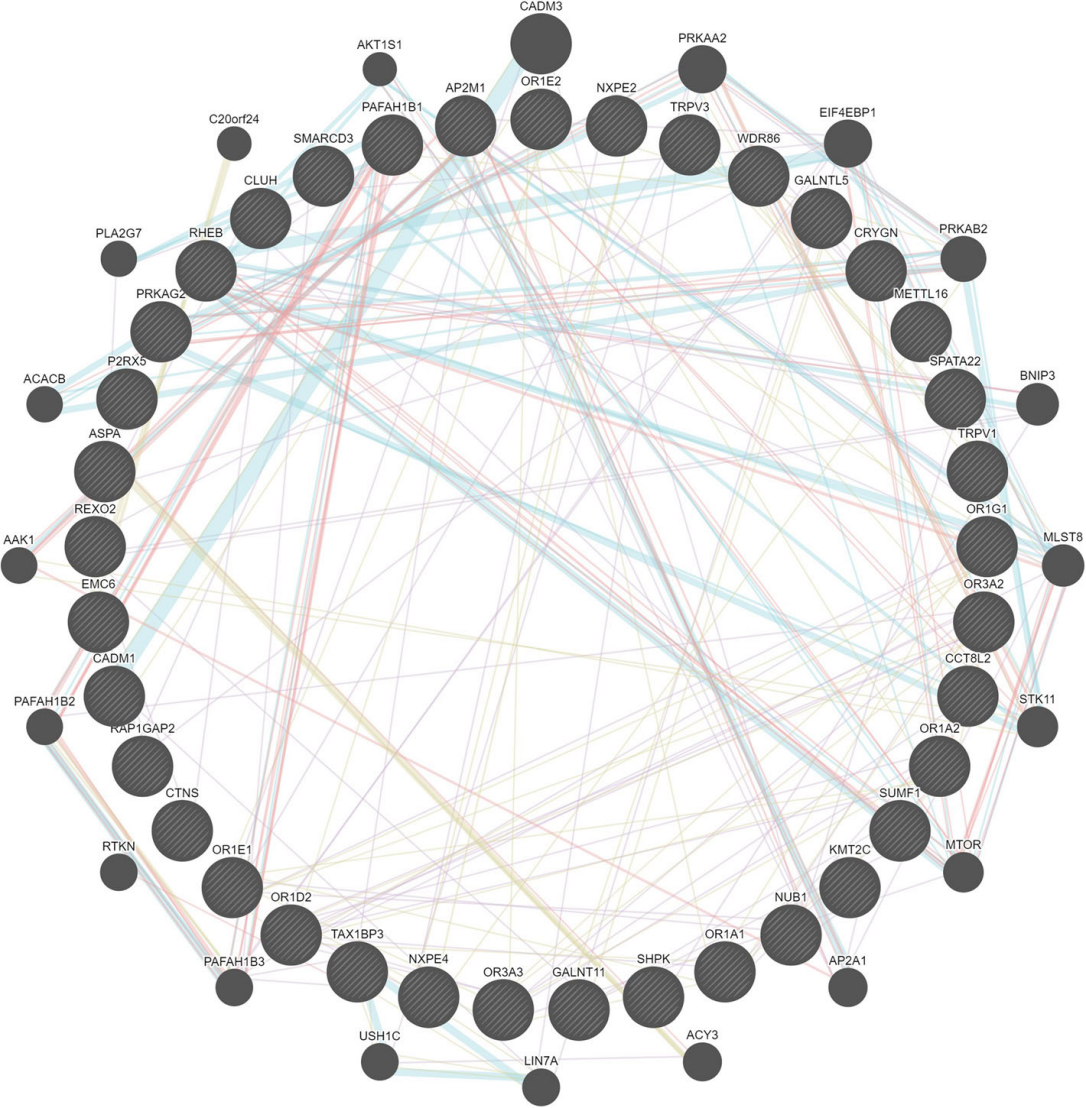
Gene networks analysis for PME per kg milk and fat traits. Dark circles with and without slash represent candidate genes and associated genes, respectively. Arrows in pink, blue, red and bone color represent co-expression, pathway, physical interactions and shared protein domains, respectively

The results of gene networks analyses for valeric acid and methane emission traits were shown in Figs. 3 and 4, respectively. Furthermore, the summary of significant SNPs (*p* < 5 × 10^− 8^) associated with PME and valeric acid traits that are in close distance to reported QTLs is presented in Table 5.

**Table 5:** QTLs located in close distance to the most significant single nucleotide polymorphisms (SNPs) associated with valeric acid and predicted methane emission (PME) per kg milk and fat traits

Trait	SNP name	QTL Trait	QTL Symbol
PME per kg fat	4:115131249	Not Available	
	19:24494923	Milk protein percentage	PP
		Milk fat yield	FY
		Milk stearic acid percentage	MFA-C18:0
		Milk conjugated linoleic acid percentage	CLAC18:2
		Milk trans-vaccenic acid percentage	MFA-C18:1 T
		Milk oleic acid percentage	MFA-C18:1
		Milk fat yield	FY
		Average daily gain	ADG
		Body weight (18 months)	BW
		Body weight (24 months)	BW
		Body weight (6 months)	BW
		Body weight (birth)	BW
		Body weight (yearling)	W365
		Residual feed intake	RFI
		Body weight (weaning)	WWT
	28:21771233	Milk yield	MY
		Body weight (mature)	MWT
		Body weight (birth)	BW
		Body weight (birth)	BW
PME per kg milk	15:25797132	Milk protein percentage	PP
		Height (mature)	MHT
Valeric acid	5:16795260	Milk fat percentage	FP
		Milk yield	MY
		Milk protein yield	PY
		Milk yield (daughter deviation)	DDMY
		Milk fat yield	FY
		Milk alpha-lactalbumin percentage	MALACTP
		Milk protein percentage	PP
		Retail product yield	YIELD
	25:37967076	Milk yield	MY
		Residual feed intake	RFI

## Discussion

The results of GWAS showed some suggestive SNPs associated with VFA traits and some regions (e.g. BTA3) associated with both acetic and propionic acid traits. Alemu et al. [11] also reported a strong correlation (− 0.85) between acetic and propionic acid. Further, we only found two SNPs significantly associated with isovaleric acid (BTA9 and 28) and 29 SNPs with valeric acid (BTA5, 11, and 25) based on the chromosome-wide Bonferroni correction threshold. The isoacids (e.g. isobutyric, 2-methylbutyric, isovaleric acid, and straight-chain valeric acid) produced in the digestion process of ruminants are mainly generated from the degradation products of the amino acids valine, isoleucine, leucine, and proline [12]. Isoacids are either required by, or stimulate the growth of ruminal cellulolytic bacteria. Therefore, they play a critical role in microbial fermentation [12]. According to the previous studies, milk production in dairy cattle could increase by 5 to 10% with the addition of isoacids to the diet [13]. Therefore, the existence of significant association between host genome and both iso-valeric acid, and valeric acid traits can help dairy farmers to improve these acids availability in rumen for ruminal cellulolytic bacteria using genetic selection.

Given that animals in this study were sampled based on highest and lowest percentiles of milk production EBV with an equal number of animals, this strategy can enhance the ability of QTL detection especially in population with small sample size. However, it has been reported that Bonferroni correction can increase the number of type II errors, particularly when the sample size is small [14]. Thus, chromosome-wide Bonferroni correction can be considered more accurate than genome-wide Bonferroni correction [15]. Since, the sample size in our GWAS was relatively small, the power of finding SNPs that are associated with PME traits was limited, and thus a few number of SNPs passed the genome-wide Bonferroni correction stringent *p*-value threshold.

Cattle have a considerable ability to produce high-quality proteins from non-edible plant cell wall components for human consumption [16]. However, it is well established that gastrointestinal microbiota contributes to feed digestion and enteric methane emission in ruminants [17–20], as approximately 17% of global methane emissions generated through ruminants [8]. Methane emission is a difficult and expensive trait to measure and is not routinely measured in dairy cattle. Furthermore, this trait has not been directly considered in the selection index of dairy cattle. However, the detrimental environmental effects of cattle production can be reduced significantly by improving the efficiency of cattle in converting feed to milk and meat, rather than wasting energy as enteric methane. To improve this efficiency in dairy cattle, it is crucial to understand the sources of genetic variation in methane production among individual animals, and the genetic architecture of methane production. van Engelen et al. [21] reported that the heritability of predicted methane yields ranged from 0.12 to 0.44 in Dutch Holstein-Friesian cows, and suggested that PME based on milk fatty acids could be reduced using genetic selection programs. de Haas et al. [7] estimated the heritability of PME and PME per fat- and protein-corrected milk yield to be 0.35 and 0.58, respectively. However, they reported that seven SNPs associated with PME in Holstein cattle could explain only 0.2% of the total genetic variance [7]. In another GWAS, 3304 significant SNPs were identified for PME (*p* < 0.005) in which 630 of them were associated with weight-at-test and dry-matter intake [8]. It has been estimated that 19 to 23% of methane emissions per kg of milk can be reduced and converted to production when cows are selected based on milk fat plus protein (19%), or based on the average genetic merit of milk fat plus protein yield (23%) [22]. According to Yan et al. [23], an effective method for mitigating methane emissions in dairy cows is to select the animals based on increased milk-production efficiency and increased energy-utilization performance.

Understanding the genetic variation and underlying genes associated with PME allows reduction of methane production in cattle through marker-assisted or genomic selection. It has been reported that genomic selection can reduce PME up to approximately 5% over 10 years [24]. The results of our study showed a large variation of PME traits indicating that mitigation of methane emissions in dairy cattle might be possible using the VFA profile of the rumen as an indicator of methane production. Moreover, reduction in methane emissions can also improve feed-conversion efficiency in dairy cattle. In addition, unlike other strategies developed to reduce methane emissions in dairy cattle such as changing the dietary formulations, feed additives, and anti-methanogen vaccines, genetic improvements have the benefits of being heritable, cumulative, and permanent.

Sixty-nine genes were identified for valeric acid (*n* = 18), PME per kg milk (*n* = 4) and PME per kg fat (*n* = 47) that were located within 1 Mb of significant SNPs. For valeric acid two of the most promising candidate genes (*ARPC1A* and *TRRAP*) were related to residual feed intake (RFI) [25, 26]. It has been observed that low-RFI pigs had a tendency toward lower valeric acid (*p* = 0.07) and isovaleric acid (*p* = 0.09) concentrations [27]. Therefore, available valeric acid concentration might be influenced by these genes in cattle. The UniProt Knowledgebase (https://www.uniprot.org/uniprot/) was used to identify the molecular functions of *ARPC1A* and *TRRAP* genes. *ARPC1A* is a component of the Arp2/3 complex, involved in regulation of actin polymerization and together with an activating nucleation-promoting factor mediates the formation of branched actin networks. *TRRAP* is an adapter protein which can be found in different multiprotein chromatin complexes with histone acetyltransferase activity that gives a specific tag for epigenetic transcription activation. Further, *TRRAP* is required for mitotic checkpoint and normal cell cycle progression. Furthermore, 17 candidate genes significantly clustered in olfactory receptors activity (GO:0004984, *p* = 4 × 10^− 10^) for PME traits. Olfactory receptors genes influenced feeding behavior such as feed intake and preferences [28]. Furthermore, they are associated with RFI and modulated olfactory transduction pathways in pig [29]. Since there was a positive genetic correlation (ranging from 0.18 to 0.84) between RFI and PME in Holstein-Friesian cows [7], olfactory receptors genes might affect methane mission per animal. In addition, five candidate genes including *CYP51A1* on BTA 4, *PPP1R16B* on BTA 13, and *NTHL1*, *TSC2*, and *PKD1* on BTA 25 suggested by Pszczola et al. [30], involved in a number of metabolic processes that might be related to methane emission. *PKD1* gene was associated with development of the digestive tract [30]. Based on the results of gene networks analyses, there were some contributions among candidate genes by co-expression, pathway, physical interactions and shared protein domains for valeric acid and methane emission traits. Most of these contributions were related to physical interactions between genes that was indicative of protein-protein interaction and if two genes showed the same protein-protein interaction, their products were linked together. Therefore, identified candidate genes in our study had a significant protein-protein interaction either together or with associated genes.

Thirty-one QTLs were identified for valeric acid (*n* = 10), PME per kg milk (*n* = 2) and PME per kg fat (*n* = 19) that were located within 1 Mb of significant SNPs. Given that methane emission is a complex trait influenced by many genes, and is not measured routinely in dairy cattle (unlike the traits of milk production and body weight), there are very limited number of QTLs associated with methane emission in the QTLdb website. Most of the QTLs that are in close distance to SNPs associated with PME traits have been found to influence milk production and its components, body weight, and RFI, rather than methane production. However, all SNPs associated with valeric acid in our study were close to the reported QTLs for milk production and its components, and RFI. The correlation coefficients between EBV for methane intensity with milk yield, fat yield, protein yield, and somatic cell score were estimated as − 0.68, − 0.13, − 0.47, and 0.07, respectively [31]. Further, a positive genetic correlation between RFI and PME (ranging from 0.18 to 0.84) was reported in Holstein-Friesian cows [7]. Herd et al. [32] also reported a high correlation between daily methane production and yearling weight (0.85), and dry-matter intake (0.79) in beef cattle. These reports indicated that some common genes and QTLs were influencing traits related to methane emission and milk production that was confirmed by our study. However, as the cows were selected based on milk production EBVs in this study, the identified QTLs and genes around significant SNPs might be increasing milk or fat production and consequently lowering methane emission per milk or fat amount. Therefore, further studies are needed to validate the results of our GWAS and investigate the biological effects of the validated SNPs on milk production and methane emission.

## Conclusions

Although 1045 SNPs passed the suggestive-association threshold (*p* < 10^− 5^) in our GWAS, only 33 of them reached the significant-association threshold (*p* < 5 × 10^− 8^) for PME per kg milk (2 SNPs); PME per kg fat (14 SNPs) and valeric acid (17 SNPs) traits. Furthermore, 17 and 40 candidate genes were discovered around the significant SNPs for valeric acid and PME (per kg milk and fat) traits. According to the GO term analysis, six promising candidate genes were clustered in organelle organization for valeric acid trait, and 17 candidate genes were clustered in olfactory receptors activity for PME trait. Moreover, the SNPs that were found in our GWAS were close to QTLs for milk yield and its components, body weight, and RFI. High correlations of these traits with PME traits and overlap of our identified genomics regions with previously reported QTLs, as well as the existence of olfactory receptors activity genes for PME traits associated with feed intake and preferences could confirm that these SNPs can be good candidates for methane emission. Overall, our findings suggest that marker-assisted and genomic selection could be used to improve the difficult and expensive-to-measure phenotypes such as PME. Moreover, prediction of methane emission by VFA indicators could be useful for increasing the size of the reference population required in GWAS and genomic selection.

## Methods

### Animals and data

Animals were selected using two-tailed strategy proposed by Jiménez-Montero et al. [33] based on the estimated breeding values (EBVs) for milk yield because there was a high negative genetic correlation (− 0.68) between milk yield EBVs and methane intensity e.g. [31], which was almost confirmed by our data set in which the phenotypic correlation between milk yield EBVs and predicted methane emission was estimated to be − 0.47. Hair and rumen digesta samples were collected from 150 Iranian Holstein cattle in a breeding population (Ferdous Pars Dairy Farm, Isfahan, Iran) in May 2016. Animals were not euthanized after the study because of the safe methods used for sampling. Estimation of breeding values were performed by National Animal Breeding Centre of Iran (Karaj, Iran) using lactation model following the Eq. 1 [34]:

1 $$ {\mathrm{y}}_{\mathrm{i}\mathrm{j}}=\mu +{\mathrm{hys}}_{\mathrm{i}}+{\mathrm{a}}_{\mathrm{i}\mathrm{j}}+{\mathrm{e}}_{\mathrm{i}\mathrm{j}} $$

where ***y***_***ij***_ is milk yield (adjusted to 305 days and twice a day milking); ***μ*** is the population mean; ***hys***_***i***_ is the effect of herd-year-season group i; ***a***_***ij***_ is the animal breeding for j^th^ animal and i^th^ herd-year-season group, and ***e***_***ij***_ is the random residual effects. The mean of the accuracy of estimated breeding values for milk yield was 0.61 [34].

The sampled animals were progeny of 42 sires and 150 dams and were born from 2011 to 2013. All animals were fed the same diet of 57.1% concentrate (barley, corn, soybean meal, lime, fish meal, meat meal, salt, fat meal, bentonite, soybean, biosaf, mineral supplement and magnesium oxide) and 42.9% forage (alfalfa, straw and corn silage) for at least two months before the sample collection. Animals were fed four times per day (morning, noon, evening and night) and had ad libitum access to water. The diet was a total mixed ration with 49% of dry matter (DM), 16.5% crude protein (60% rumen degradable protein and 40% rumen undegradable protein), 29% neutral detergent fiber, 21% acid detergent fiber, 4%lignin, 7% ash and net energy for lactation of 1.77 Mcal per kg of DM. The average amount of dry matter intake was 27.3 kg per cow per day.

### Traits studied and data collection

The rumen digesta samples were shipped to the University of Tehran’s Animal Nutrition Laboratory (Karaj, Iran) and VFA measurements were performed on these samples using the method proposed by Ottenstein and Bartley [35].

The methane emission was predicted using the Eq. 2 [36]:

2 $$ {\mathrm{CH}}_4\left(\mathrm{ml}\right)=22.4\times \left(0.5\times \mathrm{Ac}-0.25\times \Pr +0.5\times \mathrm{Bu}\right) $$

where CH_4_ is the PME, and Ac, Pr, and Bu are the concentration of acetic acid (mM), propionic acid (mM), and butyric acid (mM), respectively.

The traits include PME (ml), PME per kg milk (ml), PME per kg fat (ml), acetic acid (%), propionic acid (%), butyric acid (%), valeric acid (%) and isovaleric acid (%). The ratio of PME per cow per day to the milk or fat products per cow per day was used to estimate PME per kg product. After removing the outlier values (observations more than 3 standard deviations away from the mean), 147 samples for valeric acid and isovaleric acid, and 146 samples for the rest of the traits were remained for GWAS analyses. In our study, acetic acid (%), propionic acid (%) and butyric acid (%) had high correlation with PME (0.85, − 0.89 and − 0.14, respectively) and PME per kg of milk (0.60, − 0.62 and − 0.09, respectively) and low correlation with PME per kg of fat (0.17, − 0.17 and − 0.11, respectively).

### Genotypic data

A total of 150 cows were genotyped using the GGP-LD v4 SNP panel consists of 30,108 SNPs following the manufacturer’s protocol (GeneSeek, Nebraska, United States). The SNPs were removed from the genotyping data for call rate < 95%, minor allele frequency (MAF) < 0.05, and Chi-square < 10^− 6^ of Hardy-Weinberg equilibrium test. A total of 23,835 SNPs were retained in the analysis after the filtering. Given the markers density largely affects the power of QTL detection, imputation from BovineSNP30 panel to sequence data was carried out using stepwise imputation from the BovineSNP30 to the BovineHD beadchip (578,505 SNPs) and to sequence data (12,063,146 SNPs) [37]. The R^2^ was between 0.85 and 0.93 for imputation of high density to sequence, and between 0.65 and 0.84 for imputation of 54 k to sequence [38]. Furthermore, Van Binsbergen et al. [37] could improve the mean accuracy of imputation from 0.37 to 0.65 using the stepwise imputation (BovineSNP50 (3132 SNPs) to BovineHD (40,492 SNPs) and then to sequence data (1,737,471 SNPs) using chromosome 1 data). In the present study, stepwise imputation was carried out using Minimac3 software [39].

The 1000 Bull Genomes Project database including 129, 43, 15, and 47 key ancestors from global Holstein-Friesian, Fleckvieh, Jersey, and Angus breeds, respectively was used for imputation [10]. The same quality control was performed on sequence data, and a total of 12,063,146 SNPs were retained for the analysis after filtering. Moreover, Eagle (version 2.3) was used to phase genotypes before using Minimac3 for reference and target populations separately [40]. Finally, the imputed genotypes with accuracy lower than 0.30 were removed [41] and a total of 6,583,595 SNPs were retained for further analysis.

### Analysis of fixed effects

Data were analyzed by the least squares analysis of variance using the general linear model procedure of the SAS 9.0 (SAS, Institute Inc., Cary, NC). For analyzing PME, PME per kg milk, PME per kg fat, acetic acid, propionic acid, butyric acid, valeric acid and isovaleric acid, the contemporary group (11 levels) and age were fitted to the model as fixed effect and covariate factor, respectively.

### Genome-wide association studies

Association studies between the imputed genotypes and the phenotypes—PME (ml); PME (ml) per kg milk; PME (ml) per kg fat; and VFAs—were performed using EMMAX [42]. EMMAX simultaneously adjusted the tests for both population stratification and relatedness in the association study. The model used for GWAS is shown in Eq. 3:

3 $$ \mathbf{y}=\mathbf{X}\boldsymbol{\upbeta } +\mathbf{Zu}+\mathbf{e} $$

where **y** is the vector of phenotypic values; **X** is the design matrix relating phenotypes to their corresponding fixed effects; **β** is the vector of fixed effects (contemporary group, age, and one SNP at a time); **Z** is the design matrix relating the phenotypes to their corresponding random polygenic effects; **u** is the vector of random polygenic effects; and **e** is the vector of random residual effects. Further, we assumed that **u** ~ N (0, ${\mathbf{K}\boldsymbol{\upsigma}}_{\mathbf{g}}^{\mathbf{2}}\Big)$ and **e** ~ N (0, ${\mathbf{I}\boldsymbol{\upsigma}}_{\mathbf{e}}^{\mathbf{2}}\Big)$, where **I** is an identity matrix, and **K** is the kinship matrix constructed from sequence data.

### Significance levels

The following methods were used to determine the significance threshold of SNPs: 1) significance threshold of *p* < 5 × 10^− 8^ as proposed by Reed et al. [43]; 2) genome-wide Bonferroni correction with significance threshold of *p* < 7.59 × 10^− 9^ = *p* < 0.05 / total number of SNPs [44]; and 3) chromosome-wide Bonferroni correction with significance threshold of *p* < 0.05 / total number of SNPs on each chromosome [45]. The latter was used because Bonferroni correction would ignore the linkage between SNPs leading to conservative correction and high false-negative rate [15].

### Post-GWAS bioinformatics analysis

Ensembl annotation of UMD3.1 genome version (http://www.ensembl.org/biomart/martview) was used to identify the candidate genes and then human genes orthologous in Ensemble BioMart were identified using the gene identifiers. The DAVID Bioinformatics Resources version 6.7 (http://david.abcc.ncifcrf.gov) was used to carry out gene ontology (GO) analysis. Finally, the gene networks were drawn by GeneMANIA webserver (http://genemania.org/).

### Annotating the discovered QTL with previously reported QTL for other traits

The cattle QTLdb (https://www.animalgenome.org/cgi-bin/QTLdb/BT/index) was used to annotate QTLs within 1 Mb of the significant variants associated with the studied traits.

## Data Availability

The data set used and/or analyzed in the current study are available from the corresponding authors upon reasonable request.

## Abbreviations

PME: Predicted methane emission
VFA: Volatile fatty acid
GO: Gene Ontology
SNP: Single nucleotide polymorphism
QTL: Quantitative trait loci
GWAS: Genome-wide association studies
BTA: *Bos taurus* autosomes
RFI: Residual feed intake
EBV: Estimated breeding value
DM: Dry matter
MAF: Minor allele frequency

## References

[1] Bridgham SD, Cadillo-Quiroz H, Keller JK, Zhuang Q (2013). Methane emissions from wetlands: biogeochemical, microbial, and modeling perspectives from local to global scales. Glob Chang Biol.

[2] Cassandro M (2013). Comparing local and cosmopolitan cattle breeds on added values for milk and cheese production and their predicted methane emissions. Anim Genet Resour.

[3] Murray R, Bryant A, Leng R (1976). Rates of production of methane in the rumen and large intestine of sheep. Br J Nutr.

[4] Hristov A, Oh J, Firkins J, Dijkstra J, Kebreab E, Waghorn G (2013). Special topic--mitigation of methane and nitrous oxide emissions from animal operations: I. a review of enteric methane mitigation options. J Anim Sci.

[5] Pinares-Patiño C, Hickey S, Young E, Dodds K, MacLean S, Molano G (2013). Heritability estimates of methane emissions from sheep. Animal..

[6] Goopy JP, Donaldson A, Hegarty R, Vercoe PE, Haynes F, Barnett M (2014). Low-methane yield sheep have smaller rumens and shorter rumen retention time. Br J Nutr.

[7] De Haas Y, Windig J, Calus M, Dijkstra J, De Haan M, Bannink A (2011). Genetic parameters for predicted methane production and potential for reducing enteric emissions through genomic selection. J Dairy Sci.

[8] Manzanilla-Pech C, De Haas Y, Hayes B, Veerkamp R, Khansefid M, Donoghue K (2016). Genome wide association study of methane emissions in Angus beef cattle with validation in dairy cattle. J Anim Sci.

[9] Pickering N, Chagunda M, Banos G, Mrode R, McEwan J, Wall E (2015). Genetic parameters for predicted methane production and laser methane detector measurements. J Anim Sci.

[10] Daetwyler HD, Capitan A, Pausch H, Stothard P, Van Binsbergen R, Brøndum RF (2014). Whole-genome sequencing of 234 bulls facilitates mapping of monogenic and complex traits in cattle. Nat Genet.

[11] Alemu AW, Dijkstra J, Bannink A, France J, Kebreab E (2011). Rumen stoichiometric models and their contribution and challenges in predicting enteric methane production. Anim Feed Sci Technol.

[12] Andries J, Buysse F, De Brabander D, Cottyn B (1987). Isoacids in ruminant nutrition: their role in ruminal and intermediary metabolism and possible influences on performances—a review. Anim Feed Sci Technol.

[13] Muller LD (1987). Branched chain fatty acids (isoacids) and valeric acid for ruminants12. Prof Anim Sci.

[14] Finlay EK, Berry DP, Wickham B, Gormley EP, Bradley DG (2012). A genome wide association scan of bovine tuberculosis susceptibility in Holstein-Friesian dairy cattle. PLoS One.

[15] Han B, Kang HM, Eskin E (2009). Rapid and accurate multiple testing correction and power estimation for millions of correlated markers. PLoS Genet.

[16] Ramayo-Caldas Y, Zingaretti L, Popova M, Estellé J, Bernard A, Pons N (2019). Identification of rumen microbial biomarkers linked to methane emission in Holstein dairy cows. J Anim Breed Genet.

[17] Delgado B, Bach A, Guasch I, González C, Elcoso G, Pryce JE (2019). Whole rumen metagenome sequencing allows classifying and predicting feed efficiency and intake levels in cattle. Sci Rep.

[18] Difford GF, Plichta DR, Løvendahl P, Lassen J, Noel SJ, Højberg O (2018). Host genetics and the rumen microbiome jointly associate with methane emissions in dairy cows. PLoS Genet.

[19] Huws SA, Creevey CJ, Oyama LB, Mizrahi I, Denman SE, Popova M (2018). Addressing global ruminant agricultural challenges through understanding the rumen microbiome: past, present, and future. Front Microbiol.

[20] Tapio I, Snelling TJ, Strozzi F, Wallace RJ (2017). The ruminal microbiome associated with methane emissions from ruminant livestock. J Anim Sci Biotechnol.

[21] Van Engelen S, Bovenhuis H, Dijkstra J, van Arendonk J, Visker M (2015). Genetic study of methane production predicted from milk fat composition in dairy cows. J Dairy Sci.

[22] Bell M, Wall E, Russell G, Morgan C, Simm G (2010). Effect of breeding for milk yield, diet and management on enteric methane emissions from dairy cows. Anim Prod Sci.

[23] Yan T, Mayne C, Gordon F, Porter M, Agnew R, Patterson D (2010). Mitigation of enteric methane emissions through improving efficiency of energy utilization and productivity in lactating dairy cows. J Dairy Sci.

[24] Hayes B, Donoghue K, Reich C, Mason B, Bird-Gardiner T, Herd R (2016). Genomic heritabilities and genomic estimated breeding values for methane traits in Angus cattle. J Anim Sci.

[25] Salleh M, Mazzoni G, Höglund J, Olijhoek D, Lund P, Løvendahl P (2017). RNA-Seq transcriptomics and pathway analyses reveal potential regulatory genes and molecular mechanisms in high-and low-residual feed intake in Nordic dairy cattle. BMC Genomics.

[26] de Lima AO, de Oliveira PSN, Tizioto PC, Afonso J, Somavilla AL, da Silva J, Diniz W (2016). Association analyses pointed the TIPARP as a potential candidate gene influencing residual feed intake variation in Nelore cattle.

[27] McCormack UM, Curião T, Metzler-Zebeli BU, Magowan E, Berry DP, Reyer H (2019). Porcine feed efficiency-associated intestinal microbiota and physiological traits: finding consistent cross-locational biomarkers for residual feed intake. mSystems.

[28] Soria-Gómez E, Bellocchio L, Reguero L, Lepousez G, Martin C, Bendahmane M (2014). The endocannabinoid system controls food intake via olfactory processes. Nat Neurosci.

[29] Do DN, Strathe AB, Ostersen T, Pant SD, Kadarmideen HN (2014). Genome-wide association and pathway analysis of feed efficiency in pigs reveal candidate genes and pathways for residual feed intake. Front Genet.

[30] Pszczola M, Strabel T, Mucha S, Sell-Kubiak E (2018). Genome-wide association identifies methane production level relation to genetic control of digestive tract development in dairy cows. Sci Rep.

[31] Kandel P, Vanderick S, Vanrobays M-L, Vanlierde A, Dehareng F, Froidmont E (2014). Consequences of selection for environmental impact traits in dairy cows.

[32] Herd R, Arthur P, Bird S, Donoghue K, Hegarty R (2014). Genetic variation for methane traits in beef cattle.

[33] Jiménez-Montero JA, Gonzalez-Recio O, Alenda R (2012). Genotyping strategies for genomic selection in small dairy cattle populations. Animal..

[34] Abdollahi-Arpanahi R, Razmkabir M, Sayad Nezhad M, Eghbal A (2017). Determination of the number of test day records is required to replace lactation model with random regression model?. Iran J Anim Sci.

[35] Ottenstein D, Bartley D (1971). Improved gas chromatography separation of free acids C2-C5 in dilute solution. Anal Chem.

[36] Wolin MJ (1960). A theoretical rumen fermentation balance. J Dairy Sci.

[37] Van Binsbergen R, Bink MC, Calus MP, Van Eeuwijk FA, Hayes BJ, Hulsegge I (2014). Accuracy of imputation to whole-genome sequence data in Holstein Friesian cattle. Genet Sel Evol.

[38] Li H, Sargolzaei M, Schenkel F (2014). Accuracy of whole-genome sequence genotype imputation in cattle breeds.

[39] Das S, Forer L, Schönherr S, Sidore C, Locke AE, Kwong A, Vrieze SI, Chew EY, Levy S, McGue M (2016). Next-generation genotype imputation service and methods. Nat Genet.

[40] Loh P-R, Danecek P, Palamara PF, Fuchsberger C, Reshef YA, Finucane HK (2016). Reference-based phasing using the haplotype reference consortium panel. Nat Genet.

[41] Li Y, Willer CJ, Ding J, Scheet P, Abecasis GR (2010). MaCH: using sequence and genotype data to estimate haplotypes and unobserved genotypes. Genet Epidemiol.

[42] Kang HM, Sul JH, Zaitlen NA, Kong S-y, Freimer NB, Sabatti C (2010). Variance component model to account for sample structure in genome-wide association studies. Nat Genet.

[43] Reed E, Nunez S, Kulp D, Qian J, Reilly MP, Foulkes AS (2015). A guide to genome-wide association analysis and post-analytic interrogation. Stat Med.

[44] Nakagawa S (2004). A farewell to bonferroni: the problems of low statistical power and publication bias. Behav Ecol.

[45] Sahana G, Guldbrandtsen B, Bendixen C, Lund M (2010). Genome-wide association mapping for female fertility traits in Danish and Swedish Holstein cattle. Anim Genet.

